# Surgical outcomes for necrotizing enterocolitis in Dutch infants born before 26 weeks’ gestation

**DOI:** 10.1093/bjsopen/zraf060

**Published:** 2025-05-23

**Authors:** Otis C van Varsseveld, Adinda G H Pijpers, Ceren Imren, Joep P M Derikx, Chris H P van den Akker, Joost van Schuppen, Claudia M G Keyzer-Dekker, Marijn J Vermeulen, Maarten Schurink, Maud Y A Lindeboom, Elisabeth M W Kooi, Jan B F Hulscher

**Affiliations:** Department of Surgery, Division of Pediatric Surgery, University Medical Center Groningen, University of Groningen, Groningen, the Netherlands; Department of Pediatric Surgery, Emma Children’s Hospital, Amsterdam University Medical Centers, University of Amsterdam and Vrije Universiteit, Amsterdam, the Netherlands; Department of Pediatric Surgery, Sophia Children’s Hospital, Erasmus Medical Center, Erasmus University, Rotterdam, the Netherlands; Department of Pediatric Surgery, Emma Children’s Hospital, Amsterdam University Medical Centers, University of Amsterdam and Vrije Universiteit, Amsterdam, the Netherlands; Department of Pediatrics—Neonatology, Emma Children’s Hospital, Amsterdam Reproduction and Development Research Institute, Amsterdam University Medical Centers, University of Amsterdam and Vrije Universiteit, Amsterdam, the Netherlands; Department of Radiology and Nuclear Medicine Amsterdam UMC, University of Amsterdam, Amsterdam, the Netherlands; Department of Pediatric Surgery, Sophia Children’s Hospital, Erasmus Medical Center, Erasmus University, Rotterdam, the Netherlands; Department of Pediatric and Neonatal Intensive Care, Division of Neonatology, Sophia Children’s Hospital, Erasmus Medical Center, Erasmus University, Rotterdam, the Netherlands; Department of Pediatric Surgery, Amalia Children’s Hospital, Radboudumc, Radboud University, Nijmegen, the Netherlands; Department of Pediatric Surgery, Wilhelmina Children’s Hospital, University Medical Center Utrecht, Utrecht, the Netherlands; Division of Neonatology, Beatrix Children’s Hospital, University Medical Center Groningen, University of Groningen, Groningen, the Netherlands; Department of Surgery, Division of Pediatric Surgery, University Medical Center Groningen, University of Groningen, Groningen, the Netherlands

## Abstract

**Background:**

In infants born at < 26 weeks of gestational age (wGA) who develop necrotizing enterocolitis (NEC), medical and ethical considerations about whether surgery is the optimal treatment are complicated by a lack of group-specific outcome data. This study investigated nationwide 30-day mortality, surgical complications, and preoperative mortality risk factors in infants born at < 26 wGA who underwent surgery during the active phase of NEC.

**Methods:**

This retrospective nationwide multicentre study included all infants born at < 26 wGA undergoing surgery for Bell’s stage II/III NEC in the Netherlands between 2008 and 2022, regardless of outcome. Severe NEC was defined as Bell’s stage III (confirmed by laparotomy and/or leading to death). The primary outcome was postoperative 30-day mortality. The incidence of major postoperative complications (Clavien–Madadi III–IV) was determined after excluding infants undergoing open–close procedures for massive bowel necrosis. Potential risk factors for death after surgery were assessed using multivariable logistic regression.

**Results:**

Of 288 infants with NEC Bell’s stage ≥ II, 80 (27.8%) survived without surgery, 66 (22.9%) died before laparotomy, and 142 (49.3%) underwent laparotomy. In 142 surgically treated infants with severe NEC (57.0% male), the median gestational age was 25 + 0 (range 23 + 6 to 25 + 6) weeks + days, the median birthweight was 750 (range 485–1070) g, and the median age at surgery was 14 (range 2–66) days. Primary open–close surgery was performed in 34 of 142 infants (23.9%). In the remaining 108 infants, surgical management included stoma creation (63.0%), primary anastomosis (27.8%), or both (9.3%). Overall, the 30-day mortality rate among 142 infants was 47.2% (67 deaths). Death occurred after a primary or second-look open–close procedure in 37 infants, after multiorgan failure in 17, and from other causes in the remaining 13. After excluding 37 infants who died after open–close procedures, 30-day complications occurred in 23 (21.9%) of 105 surgically treated infants. There were 29 events in total, including reoperation for bowel perforation (5, 17%) or anastomotic leak/stenosis (5, 17%). Regression analysis identified no risk factors for 30-day mortality.

**Conclusion:**

The 30-day mortality rate was 47.2% in infants born at < 26 wGA undergoing NEC surgery, most of whom died after an open–close procedure. Another 21.9% of infants experienced major complications.

## Teaser

This study assessed outcomes of necrotizing enterocolitis (NEC) surgery in a nationwide Dutch population of 142 infants born before 26 weeks gestation. The 30-day mortality rate was 47.2%, with more than half of deaths (55%) attributable to an open–close procedure for massive bowel necrosis. Major complications occurred in 21.9% of infants. The study findings could be helpful for parent counselling, decision-making, and guideline development in extremely preterm infants who develop severe NEC.

## Introduction

Necrotizing enterocolitis (NEC) is a life-threatening gastrointestinal disease that primarily affects preterm or low-birthweight infants^1,2^. NEC is the leading cause of mortality and morbidity from gastrointestinal diseases in preterm infants, with an estimated incidence of 3–17% in those with very low birthweight (< 1500 g)^1–6^. Approximately one in three cases of NEC is lethal, with infants of lower birthweight and lower gestational age faring even worse^1,3,6^. Surgery is performed in approximately 60% of infants with NEC^6–8^, with a greater need for surgery in the most extremely preterm infants^9,10^.

During NEC surgery, the necrotic intestine is typically resected, after which an anastomosis or stoma is constructed^1,2^. Within the first month after surgery, mortality rates reach over 50%, emphasizing the severity of the disease^3,6,11^. Of those infants who survive after NEC surgery, up to one-quarter experience gastrointestinal sequelae, and over 40% experience neurodevelopmental delay^11–13^. Hence, each case of NEC in a critically ill infant presents both the medical team and the parents with an urgent medical–ethical dilemma as to whether NEC surgery is still in the best interest of the infant, or whether comfort care (namely palliative care, resulting in death) should be considered^14,15^.

Advances in neonatal intensive care, allowing resuscitation of more extremely preterm infants, have contributed to the increased incidence of NEC^1,4,16^. In the Netherlands, since 2010, guidelines have prescribed active resuscitation of infants born at 24 + 0 weeks of gestational age (wGA) or later, after parental consent^1,17^. This recommendation remained applicable throughout the duration of the present study. Despite advances in neonatal care, contemporary mortality and morbidity rates for NEC have remained unchanged, both in the Netherlands and globally^1,18^. Current literature mostly focuses on the outcomes of the broader (surgical) NEC population^18^, but there is limited knowledge regarding outcomes specifically in extremely preterm infants born at < 26 wGA. In addition, the number of infants being redirected to comfort care despite having an indication for surgery is often disregarded, although this is an important contextual factor for interpreting surgical outcomes in this specific subgroup. In this vulnerable group of the smallest, extremely preterm infants in particular, the decision to proceed with surgery is complex and context-dependent.

Group-specific outcome data of surgical NEC in extremely preterm infants is paramount for optimal parental counselling, shared decision-making, and guideline development in critical situations where surgical intervention may pose a dilemma. Hence, the primary aim of the present study was to determine 30-day mortality in a nationwide cohort of extremely preterm infants who were treated surgically for NEC. Secondary aims were to analyse 30-day complication rates, postoperative surgical complications, changes in surgical outcomes over time, and preoperative risk factors for 30-day mortality. The number of infants receiving comfort care was assessed for contextual interpretation of this selected surgical population.

## Methods

### Study design and patients

A nationwide multicentre retrospective cohort study was conducted in five Dutch academic medical centres with level IV neonatal intensive care units (NICUs) and in-house paediatric surgical services: University Medical Center Groningen (UMCG), Amsterdam University Medical Centers (AUMC; locations University of Amsterdam and Vrije Universiteit), Erasmus Medical Center, University Medical Center Utrecht, and Radboud University Medical Center. In the participating centres, infants born between 24 and 26 wGA are eligible for active resuscitation, with those born 1 day below this lower limit being eligible for resuscitation only sporadically. The decision to administer active resuscitation is made by the parents in consultation with the medical team, considering individual prognostic factors and parental preferences.

The requirement for ethics approval for this study was waived by the Institutional Review Board of the UMCG (CTc 201800771), based on the Dutch Medical Research Involving Human Subjects Act. In the AUMC and UMCG, a general consent opt-out approach was used (W18_233#18.278 and CTc 201800771, respectively). In other participating centres the requirement for informed consent was waived (MEC-2013-409, 20-182/C, and 2020-6365) because of the observational nature of the study.

This study included all infants born before 26 + 0 wGA between 1 January 2008 and 31 December 2022 who developed severe NEC and underwent surgery for NEC during the active phase of the disease. Clinically, NEC was diagnosed based on a combination of clinical signs (abdominal distension, bilious gastric aspirates, and/or rectal blood loss) and radiological signs (pneumatosis intestinalis, portal venous gas, and/or pneumoperitoneum on abdominal X-ray). Surgery during the active phase of NEC was defined as within 10 days after clinical diagnosis, provided that the infant had ongoing clinical and/or radiological signs of NEC up to the time of surgery. The NEC stage was subsequently determined based on clinical, radiological, and surgical findings, in accordance with the modified Bell’s staging^19^, selecting infants with NEC stage II/III. For this study, severe NEC was defined as a disease confirmed by laparotomy, histology, or autopsy, or, if no tissue evidence was available, the reported primary cause of death in the discharge letter^20^. This definition retrospectively denotes all infants who died from NEC without laparotomy (preoperative diversion to palliative care) and those who underwent laparotomy for NEC. By definition, this excluded infants with successful medically treated NEC. The primary study population included only infants who underwent laparotomy for severe (stage III) NEC (regardless of outcome), whereas this paper reports the number of infants who died before surgery from severe NEC (palliation) and those with medically treated NEC for context. Infants were excluded if they had a focal/spontaneous intestinal perforation. This was defined as intraoperative identification of an isolated perforation without adjacent inflamed and/or ischaemic bowel segments, corroborated by the radiological absence of pneumatosis intestinalis and the histopathological absence of transmural inflammation and ischaemia in bowel tissue adjacent to the perforation (if available)^21^.

### Outcomes of interest

The primary outcome of the study was 30-day mortality, defined as up to and including 30 days after the first surgical intervention for severe (stage III) NEC^22^. Secondary outcomes were 30-day major complication rates, postoperative surgical complications up to the last surgical follow-up, change in surgical outcome over time, and preoperative risk factors for 30-day mortality. In addition, the full population of infants born at < 26 wGA treated for NEC stage II and III was assessed to provide the proportion of each type of management applied (medical, surgical, palliative) as a reference for interpreting the clinical selection bias in the surgical NEC population.

### Data collection and definitions

All data were collected retrospectively from medical records by the authors. Data were cross-validated between centres by 10% random samples and descriptive statistics screening. When the diagnosis of NEC was doubted, it was discussed with a supervising paediatric surgeon and/or neonatologist.

Demographic data included gestational age, birthweight, sex, antenatal steroid administration, mode of delivery (caesarean section or vaginal), Apgar score at 5 min, cardiac anomalies^23^, and intraventricular haemorrhage (IVH; Papile grade 1–3, with or without infarction)^24^. Major cardiac anomalies were defined as any anomaly necessitating surgery; all other cardiac anomalies were considered minor. Patent foramen ovale and patent ductus arteriosus were not recorded as cardiac anomalies^23,25^. Disease course characteristics recorded included cardiovascular support, radiological findings (based on the radiology report), NEC stage, time of NEC diagnosis and surgery, length of NICU stay, and follow-up duration. A review of radiological images was only performed for infants with an ambiguous NEC diagnosis description.

Intraoperative data were extracted from surgical reports and included the location of the intestinal perforation, the presence and aspect of ascites, the mode of treatment, and the length and location of the bowel resection. NEC totalis was defined as a degree of small bowel necrosis that was deemed incompatible with a reasonable quality of life by the surgical team^26^. In these circumstances, an open–close procedure was performed, where no resection or other intervention was undertaken and the infant was diverted to comfort care, resulting in death.

Thirty-day postoperative complications were classified according to the Clavien–Madadi (CM) classification, a five-level grading system designed and validated specifically for paediatric surgical complications^27^. For this study, non-lethal major postoperative complications, defined as CM grade III–IV, were assessed and the cause of death (CM grade V) was described separately. CM grade III complications led to unplanned reintervention, and grade IV complications led to multiorgan dysfunction (dysfunction of 2 or more organs or systems: respiratory, cardiovascular, haematological, neurological, gastrointestinal, hepatic, renal)^27^.

Postoperative surgical complications assessed included stoma revision surgery, anastomotic leak, anastomotic stricture, adhesion ileus, fascial dehiscence, and post-NEC stenosis. In addition, unplanned reoperations, the total number of abdominal surgeries, and the cause of death were assessed^12^. Adhesion ileus was confirmed upon reoperation for bowel obstruction^28^. Post-NEC stenosis was defined as a clinically relevant intestinal stenosis, based on obstructive symptoms including the inability to increase enteral feeding and/or absence of defaecation for > 48 hours (h) with the need to perform a contrast enema study and/or surgery, all after the active phase of NEC^29^. When strictures were diagnosed as located on the former anastomotic site, these were regarded as anastomotic strictures. Stoma reversal was not considered a stoma revision, and anastomotic leak and stricture after stoma reversal were scored separately. Planned second-look surgeries were not counted as unplanned reoperations^30^. Despite not fulfilling the criteria for a major complication (CM grade ≥ III), high-output stoma was recorded separately because of its clinical relevance and was defined as physician-reported excessive stoma output leading to a change in medical course (that is, an inability to reach full enteral feeding, earlier than planned stoma closure)^7^.

The time to full enteral feeding (in days), based on the cessation of parenteral feeding, was also recorded and related to short bowel syndrome and intestinal failure. Short bowel syndrome was defined in accordance with Olieman *et al*.^31^ as a > 70% resection of the small bowel and/or parenteral nutrition needed for longer than 42 days after bowel resection. Intestinal failure was defined as the need for parenteral nutrition for ≥ 90 days after bowel resection^32,33^. For each centre, the number of infants born at < 26 wGA with NEC stage ≥ II was retrieved, as was the number of these infants receiving either: medical management for NEC (‘medically treated NEC’, defined as maximum therapeutic or supportive medical treatment without surgical intervention); surgical intervention for severe NEC (‘surgical NEC’, defined as any surgical intervention for NEC from drainage to laparotomy); or diversion to palliative care for severe NEC (‘comfort care’) without undergoing surgery for NEC.

### Statistical analysis

Normally distributed continuous variables are reported as the mean with standard deviation (s.d.), whereas skewed variables are reported as the median with range (minimum to maximum). Data distribution was analysed using histograms. Statistical significance was defined as a two-tailed *P* < 0.050. Because of the explorative nature of the study, no correction for multiple testing was applied. To gain a greater insight into the 30-day postoperative course, a 30-day Kaplan–Meier survival function was plotted.

Logistic regression analysis was used to identify risk factors for 30-day mortality. Potential preoperative risk factors were selected based on a literature search and clinical relevance. Collinearity between variables was assessed by Pearson’s *r* (parametric data) and Spearman’s ρ (non-parametric data), wherein a correlation coefficient of ≥ 0.5 was considered significant, leading to the exclusion of a variable from univariable logistic regression analysis^34^. The univariable analysis included the following preoperative factors: mode of delivery, gestational age at birth, birthweight, sex, IVH, postnatal age at NEC diagnosis, cardiovascular support (inotropes and vasopressors) between NEC diagnosis and surgery, and pneumoperitoneum on radiological imaging^35–40^. Forward selection was used to construct a multivariable model, including those variables with a univariable logistic regression *P* ≤ 0.250^41^. For variables included in the regression analysis, there were no missing data and no imputations were performed.

To evaluate the time trend over the study period of 15 years, a time variable (year of first NEC operation) was included in the univariable and multivariable analyses. Based on graphic evaluation of the outcomes over time, no non-linear terms were deemed necessary. Risk factors are reported as an odds ratio (OR) with 95% confidence interval (c.i.). All analyses were conducted using SPSS^®^ version 28 (IBM, Armonk, NY, USA).

## Results

### Demographics

**Fig. 1:**
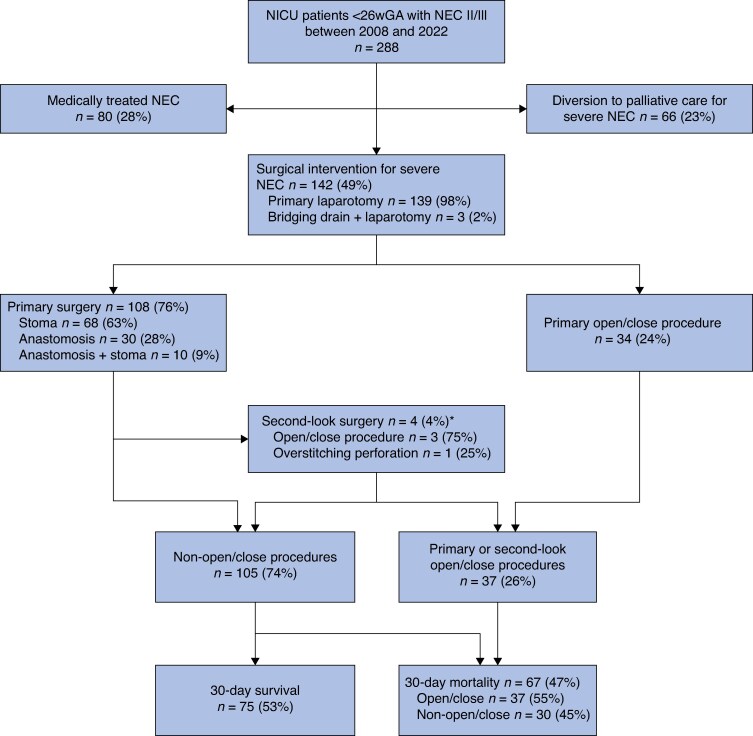
Flow chart of inclusion and disease course for infants with NEC stage II/III *Four infants from the primary surgery group of 108 underwent a planned second-look surgery 24–72 hours after the primary surgery; 3 of these resulted in an open–close procedure and 1 entailed overstitching of a perforation, increasing the total of open–close procedures to 37 and reducing the total of non-open–close procedures to 105. NEC, necrotizing enterocolitis; NICU, neonatal intensive care unit.

Overall, 288 infants born at < 26 wGA were identified and treated for NEC stage II/III between 2008 and 2022 (*Fig. 1*). Data on surgical NEC were complete for all 15 study years in all 5 centres, whereas data specifically on medical NEC and/or comfort care were incomplete for the study years 2008–2012 in 1 centre and for 2018 in another centre. Of the 288 infants identified, 80 (27.8%) were treated medically for NEC, 142 (49.3%) underwent surgical intervention for severe NEC, and 66 (22.9%) were diverted to comfort care before surgery for severe NEC. Of the 142 infants who underwent surgery for severe NEC during the active phase of the disease, 81 were male (57.0%) and 56 (39.4%) were born at < 25 wGA (*Table 1*). The median gestational age was 25 + 0 (range 23 + 6–25 + 6) weeks + days and the median birthweight was 750 (range 485–1070) g. Most of the 142 infants were born by vaginal delivery (101, 71.1%).

**Table 1:** Patient characteristics

	Entire cohort (*n* = 142)	Patients who died within 30 days after surgery (*n* = 67)	Patients who died within 30 days after open–close surgery (*n* = 37)
**Sex**			
Male	81 (57.0%)	42 (63%)	27 (73%)
Female	61 (43.0%)	25 (37%)	10 (27%)
Gestational age (weeks + days), median (range)	25 + 0 (23 + 6–25 + 6)	25 + 0 (23 + 6–25 + 6)	25 + 0 (24 + 0–25 + 6)
Gestational age < 25 weeks	56 (39.4%)	26 (39%)	15 (41%)
Birthweight (g), median (range)	750 (485–1070)	750 (545–1070)	750 (585–1070)
Fenton birthweight z-score, median (range)	0.20 (−2.10 to 3.10)	0.20 (−1.30 to 2.24)	0.20 (−1.30 to 2.24)
**Antenatal steroids**			
Partial (1 dose)	26 (18.3%)	15 (22%)	10 (27%)
Complete (2 doses)	78 (54.9%)	33 (49%)	18 (49%)
Vaginal delivery	101 (71.1%)	48 (72%)	29 (78%)
Multiple birth	46 (32.4%)	27 (40%)	12 (32%)
Apgar score at 5 min	7 (2–10)	7 (2–10)	7 (2–9)
Minor cardiac anomaly	2 (1.4%)*	0 (0%)	0 (0%)
**IVH**			
No IVH	75 (52.8%)	36 (54%)	23 (62%)
Grade I–II	52 (36.6%)	22 (33%)	10 (27%)
Grade ≥ III	15 (10.6%)	9 (13%)	4 (11%)
Cardiovascular support in the first week of life	42 (29.6%)	22 (33%)	15 (41%)
**Stage of NEC**			
IIIA	42 (29.6%)	16 (24%)	11 (30%)
IIIB†	100 (70.4%)	51 (76%)	26 (70%)
Postnatal age at diagnosis (days), median (range)	13 (2–66)	11 (2–66)	9 (2–55)
Time between diagnosis and surgery (days), median (range)	1 (0–10)	1 (0–9)	1 (0–6)
CV support between NEC diagnosis and surgery	75 (52.8%)	40 (60%)	25 (68%)
**Radiological findings**			
Pneumatosis intestinalis	93 (65.5%)	45 (67%)	26 (70%)
Pneumoperitoneum	71 (50.0%)	38 (57%)	21 (57%)
Portal gas	25 (17.6%)	14 (21%)	9 (24%)
Postnatal age at surgery (days), median (range)	14 (2–66)	12 (3–66)	10 (3–56)
Operated on in NICU	59 (41.5%)	34 (51%)	19 (51%)
**Primary mode of treatment**‡			
Primary anastomosis	30 (21.1%)	6 (9%)	-
Stoma creation	68 (47.9%)	24 (36%)	2 (5%)
Both (anastomosis and stoma)	10 (7.0%)	3 (5%)	1 (3%)
Open–close procedure (NEC totalis)	34 (23.9%)	34 (51%)	34 (92%)
**Planned second-look procedure**			
Overstitch perforation	1 (1.4%)		
Open–close procedure (NEC totalis)	3 (2.1%)	3 (4%)	3 (8%)
30-day postoperative mortality	67 (47.2%)	67 (100%)	37 (100%)
Age at death (days), median (range)	20 (3–337)	15 (3–71)	10 (3–59)
Total length of NICU stay (days), median (range)	46 (2–175)	13 (2–67)	10 (2–47)
Duration of follow-up (months), median (range)	1 (0–163)	0 (0–0)	0 (0–0)

*Included a minor stenosis of the left pulmonary veins and a minor muscular ventricular septum defect. †Stage IIIB included 28 of 100 infants (28.0%) in whom no pneumoperitoneum was detected on abdominal X-ray, but intestinal perforation was encountered at surgery—typically in contained perforation or when perforation was only identified by supplementary ultrasonography. It also included 18 of 100 infants (18.0%) in whom pneumoperitoneum was suspected on abdominal X-ray but any mention of a perforation (or not) was omitted from the surgical report and no pathological report was retrievable—mostly in infants undergoing open–close procedures, wherein no further bowel exploration was conducted. ‡Three infants had a bridging drain before the primary laparotomy in which one underwent stoma creation, one underwent primary anastomosis, and one underwent an open–close procedure. IVH, intraventricular haemorrhage; NEC, necrotizing enterocolitis; CV, cardiovascular; NICU, neonatal intensive care unit.

### Disease onset

The median postnatal age at NEC diagnosis was 13 (range 2–66) days. Of the 142 infants who underwent surgery for severe NEC, 42 (29.6%) received cardiovascular support in their first week of life and 75 (52.8%) received cardiovascular support between NEC diagnosis and surgery. The most common radiological finding was pneumatosis intestinalis in 93 infants (65.5%), followed by pneumoperitoneum in 71 (50.0%), and portal gas in 25 (17.6%). There were no conclusive radiological signs of NEC before explorative laparotomy in 19 infants (13.4%). All infants had a modified Bell stage of III: stage IIIA in 42 (29.6%) and stage IIIB in 100 (70.4%).

### Surgical management

The median time between NEC diagnosis and surgery was 1 (range 0–10) day and the median age at surgery was 14 (2–66) days. Of the 142 infants who underwent surgery for severe NEC, 3 (2.1%) had a bridging peritoneal drainage before the primary laparotomy. A bowel perforation was encountered during surgery in 80 infants (56.3%), most frequently of the ileum (31 of 80, 39%) (*Table 2*). Thirty-four infants (23.9%) underwent a primary open–close procedure without bowel resection; the extent of small bowel necrosis was deemed incompatible with a reasonable quality of life by the surgical team.

**Table 2:** Intraoperative characteristics

	No. of patients* (*n* = 142)
Ascites	78 (54.9%)
**Type of ascites (*n* = 78)**	
Faecal	41 (53%)
Clear/serous	18 (23%)
Purulent	11 (14%)
Unspecified	8 (10%)
Bowel perforation found	80 (56.3%)
**No. of perforations (*n* = 80)**	
1	49 (61%)
2	13 (16%)
≥ 3	7 (9%)
Unspecified	11 (14%)
**Location of perforation (*n* = 80)**	
Jejunum	10 (12%)
Ileum	31 (39%)
Small bowel (unspecified)	14 (18%)
Colon	14 (18%)
Small bowel and colon	7 (9%)
Unspecified	4 (5%)
**Bowel resection**	108 (76.1%)
Resected bowel length (cm), median (range)†	15 (1–75)
Ileocecal resection	31 (22%)
Histological confirmation of NEC‡	109 (76.8%)
Stoma at primary surgery	78 (54.9%)
Proximal stoma (< 50 cm of Treitz) (*n* = 78)	34 (44%)
Anastomosis at primary surgery	40 (28.1%)
No. of anastomoses (*n* = 40)	
1	34 (85%)
2	4 (10%)
4	2 (5%)

*Unless otherwise indicated. †Based on the combined segment lengths described in the surgical report; data were missing for 10 of 108 infants (7%) who underwent resection. ‡Histological confirmation was available for 108 infants through a resected bowel segment and for one infant through autopsy; 33 infants with necrotizing enterocolitis (NEC) totalis without bowel resection or autopsy were only confirmed by laparotomy.

Of the 108 infants who underwent bowel resection, the primary mode of treatment was stoma creation (68, 63.0%), primary anastomosis (30, 27.8%), or a combination (10, 9.3%). Four infants (3.7%) had a planned second-look laparotomy 24–72 h after the primary procedure. In 3 of these infants, extensive bowel necrosis led to an open–close procedure, increasing the total of open–close procedures among the 142 infants overall to 37 (26.1%) and reducing the total of non-open–close procedures to 105 (73.9%). One second-look laparotomy involved overstitching of a perforation.

### Thirty-day mortality

**Fig. 2:**
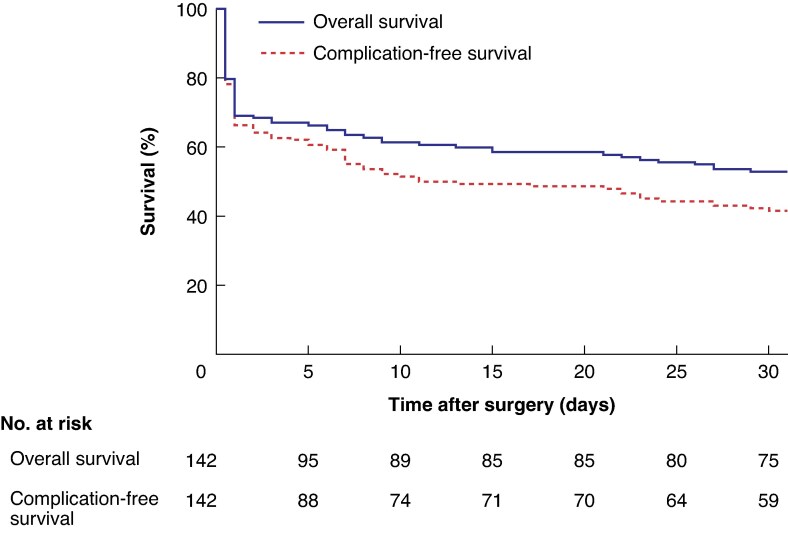
Kaplan–Meier 30-day survival curves for the full cohort of 142 infants

Among the 142 infants overall, the 30-day mortality rate was 47.2% (67 deaths), with 44 of these infants (66%) dying within 1 day after surgery (*Fig. 2*). Of the 67 infants who died within 30 days after surgery, 37 (55%) underwent open–close procedures.

In the remaining 30 infants who died, the causes of death were multiorgan failure (17, 57%), intensive care withdrawal due to an unfavourable prognosis—including a poor neurological prognosis (7, 23%) or extensive bowel necrosis upon unplanned reoperation (4, 13%)—and intra-abdominal bleeding after surgery (2, 7%) (*Table 3*). For reference, over the full < 26 wGA population with severe NEC (208 infants), the mortality rate was 63.9% (133 deaths), comprising 66 infants (49.6%) palliated before surgery, 37 (27.8%) palliated after open–close surgery due to NEC totalis, and 30 (22.6%) who died after operation despite bowel resection.

**Table 3:** Thirty-day postoperative complications in 105 infants who did not undergo a primary or second-look open–close procedure and causes of death of 67 infants

	*n* (%)
Non-lethal major complications (CM III–IV) at 30 days (*n* = 105)*	
No. of events	29
No. of infants	23 (21.9%)
Complications (*n* = 29)	
(Sepsis with) multiorgan failure	5 (17%)
Reoperation for anastomotic stricture or leak	5 (17%)
Reoperation for bowel perforation	4 (14%)
Severe periventricular or intraventricular haemorrhage	2 (7%)
Reoperation for recurrent NEC	2 (7%)
Central line placement in operating theatre	2 (7%)
Others†	9 (31%)
Relaparotomy for a major (CM III-IV) complication	23 (21.9%)
30-day mortality (*n* = 142)	67 (47.2%)
**Causes of death (CM V) (*n* = 67)**	
Primary open–close surgery	33 (49%)
Open–close procedure upon planned second look	4 (6%)
Multiorgan failure	17 (25%)
IC withdrawal due to poor neurological prognosis	7 (10%)
IC withdrawal due to extensive bowel necrosis upon unplanned reoperation	4 (6%)
Intra-abdominal bleeding after surgery	2 (3%)

*Includes 7 non-lethal major complications in the 30 deceased infants who did not undergo an open–close procedure: 2 with (sepsis with) multiorgan failure, 2 with severe periventricular or intraventricular haemorrhage, 2 with anastomotic leak, and 1 with progression of bowel necrosis. †Includes singular, non-lethal cases of complicated wound infection, adhesion ileus, fascial dehiscence, progressive bowel necrosis, post-necrotizing enterocolitis (NEC) stenosis, explorative relaparotomy for clinical deterioration, cardiopulmonary resuscitation, ultrasound-guided intervention, and incisional hernia. CM, Clavien–Madadi grades^27^; IC, intensive care.

### Thirty-day surgical complications

Infants who underwent an open–close procedure were excluded from the surgical complications overview because they did not undergo bowel resection and died shortly after surgery. Major 30-day postoperative complications (CM III–IV) occurred in 23 of 105 infants (21.9%). Some infants had two major complications, so there was a total of 29 events (*Table 3*). Of the 29 events recorded, the most frequent major complications were multiorgan failure (5, 17%), reoperation for anastomotic stenosis or leak (5, 17%), and reoperation for bowel perforation (4, 14%). In total, 23 of 105 infants (21.9%) underwent relaparotomy for a major complication. When including cause of death (CM V), the combined major complication and mortality rate (CM III–V) was 43.8% (46 deaths) among the 105 infants who did not undergo an open–close procedure. In the full cohort (142 infants), the rate of major complication-free survival beyond 30 days was 41.5% (59 infants) (*Fig. 2*).

### Complications up to the last surgical follow-up

The median surgical follow-up in infants who did not undergo an open–close procedure was 7 (range 0–163) months (*Table 4*). Within the stoma creation group (78 infants), 18 (23%) high-output stomas occurred and 1 infant (1%) underwent stoma revision surgery. Within the primary anastomosis group (40 infants), 6 infants (15%) developed an anastomotic leak and 8 (20%) developed an anastomotic stricture. Four infants (10%) with a primary anastomosis had a stoma created during an unplanned reoperation: one due to recurrent NEC, two because of an anastomotic leak, and one because of post-NEC stenosis. One of these infants developed a high-output stoma.

**Table 4:** Postoperative course up to the last surgical follow-up in infants who did not undergo a primary or second-look open–close procedure

	No. of patients* (*n* = 105)
Duration of follow-up (months), median (range)	7 (0–163)
Total length of NICU stay (days), median (range)	73 (6–175)
Fascial dehiscence	6 of 105 (5.7%)
Adhesion ileus	6 of 105 (5.7%)
Post-NEC stenosis	5 of 105 (4.8%)
High-output stoma	18 of 78 (23%)†
Stoma revision surgery	1 of 78 (1%)†
Anastomotic leak**	6 of 40 (15%)‡
Anastomotic stricture**	8 of 40 (20%)‡
Stoma creation at reoperation	4 of 40 (10%)‡
Time to full enteral feeding (days), median (range)	76 (8–320)
Short bowel syndrome	24 of 105 (22.9%)
Intestinal failure	17 of 64 (27%)§
**Stoma reversal**	51 of 55 (93%)∫
Time to stoma reversal (days), median (range)	75 (39–360)
Age at stoma reversal (months), median (range)	3.2 (2.1–13.4)
Anastomotic leak after reversal††	1 of 51 (2%)
Anastomotic stricture after reversal‡‡	3 of 51 (6%)
**Total no. of abdominal surgeries**	
1	34 of 105 (32.4%)
2	44 of 105 (41.9%)
≥ 3	27 of 105 (25.7%)

*Unless otherwise indicated. †The denominator includes all 78 infants with stoma creation at primary surgery. ‡The denominator includes all 40 infants with primary anastomosis at primary surgery. §The denominator includes all infants surviving up to 90 days after surgery^32,33^. ∫Fifty-five infants survived beyond 30 days, four of whom died before undergoing stoma reversal. **Using the absolute number of anastomoses made (50) instead of the number of infants, the leak rate was 12.0% (6) and the stricture rate was 16.0% (8). ††Treated conservatively. ‡‡Two infants were treated by incision and restitching, and one by resection and new hand-sewn anastomosis. NICU, neonatal intensive care unit.

Fifty-five infants with a stoma survived beyond 30 days, of whom 51 (93%) had stoma reversal at a median of 75 (range 39–360) days after stoma creation and a median age of 3.2 (range 2.1–13.4) months. Four infants (7%) died before undergoing stoma reversal. After stoma reversal, one anastomotic leak (2%) and three anastomotic strictures (6%) occurred. Within the whole cohort, 64 infants (45.1%) survived up to 90 days after surgery, of whom 17 (27%) had intestinal failure.

### Thirty-day mortality risk factors

**Fig. 3:**
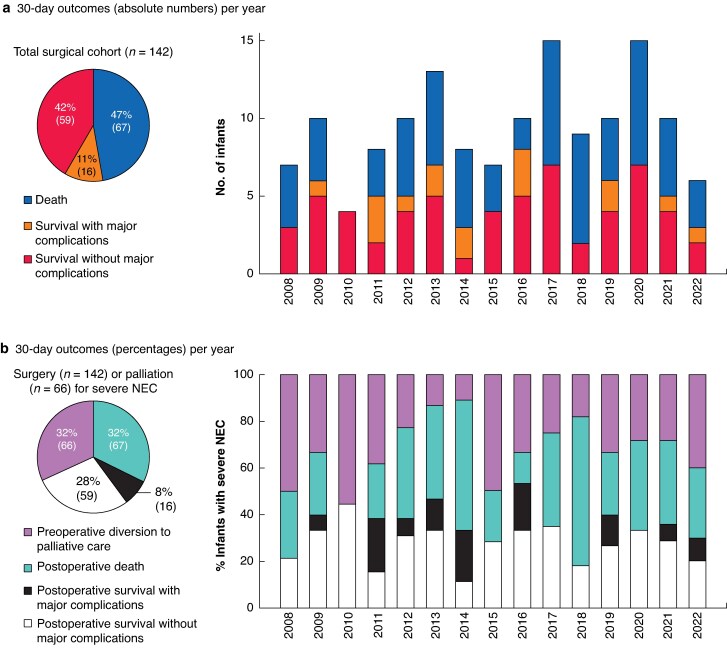
Thirty-day outcomes in infants with surgically treated severe NEC **a** Thirty-day outcomes in absolute numbers per year. **b** Thirty-day outcomes, including preoperative diversion to palliative (comfort) care, in relative percentages per year, with absolute numbers within the stacked bars. NEC, necrotizing enterocolitis.

Time (year of first NEC operation) had no effect on the odds of open–close procedures (OR 1.00, 95% c.i. 0.91 to 1.10; *P* = 0.977), 30-day major complications (OR 0.99, 0.89 to 1.10; *P* = 0.844), 30-day mortality (OR 1.04, 0.96 to 1.13; *P* = 0.365), or a combination of 30-day mortality and major complications (OR 1.00, 0.93 to 1.09; *P* = 0.925) (*Fig. 3*). Four potential preoperative risk factors for 30-day mortality had *P* values below the predetermined threshold of 0.250 in the univariable analysis, namely: postnatal age at NEC diagnosis (OR 0.98, 0.96 to 1.00; *P* = 0.100), with lower age at higher risk; sex (OR 1.55, 0.79 to 3.03; *P* = 0.200), with males at higher risk; cardiovascular support between NEC diagnosis and surgery (OR 1.69, 0.87 to 3.30; *P* = 0.121); and pneumoperitoneum (OR 1.67, 0.86 to 3.24; *P* = 0.131) (*Table 5*). These four factors were included in the multivariable model. However, in multivariable logistic regression analysis, none of these factors remained as a statistically significant preoperative risk factor for 30-day mortality.

**Table 5:** Risk factors for 30-day mortality (67 deaths) in univariable and multivariable logistic regression analyses

	Univariable analysis		Multivariable analysis*	
	Odds ratio	*P*	Odds ratio	*P*
Vaginal birth	1.05 (0.51, 2.17)	0.100		
Gestational age at birth	0.98 (0.89, 1.07)	0.596		
Birthweight per 50 g	1.00 (0.87, 1.15)	0.958		
Male sex	1.55 (0.79, 3.03)	0.200	1.39 (0.69, 2.77)	0.355
IVH ≥ grade II with infarction	1.78 (0.60, 5.31)	0.298		
Postnatal age at NEC diagnosis	0.98 (0.96, 1.00)	0.100	0.99 (0.96, 1.02)	0.471
CV support between NEC diagnosis and surgery	1.69 (0.87, 3.30)	0.121	1.52 (0.76, 3.03)	0.232
Pneumoperitoneum on radiological imaging	1.67 (0.86, 3.24)	0.131	1.37 (0.64, 2.94)	0.415
Year of first NEC surgery	1.04 (0.96, 1.13)	0.365	1.02 (0.94, 1.11)	0.696

Values in parentheses are 95% confidence intervals. IVH, intraventricular haemorrhage; NEC, necrotizing enterocolitis; CV, cardiovascular. *Risk factors with *P* ≤ 0.250 in univariable logistic regression analysis were included in the multivariable model, supplemented with the year of first NEC surgery.

## Discussion

The present nationwide Dutch cohort study found a 30-day mortality rate of 47.2% in infants born at < 26 wGA who underwent surgery for severe NEC; approximately half these deaths (55%) were attributable to the presence of extensive NEC for which an open–close procedure was performed. Non-lethal major 30-day complications occurred in another 22% of infants. Surgical outcomes did not change significantly over the 15-year study period. No independent risk factors for 30-day mortality were found. Of all infants born at < 26 wGA who were diagnosed with severe NEC, 31.7% received comfort care.

In previous US and British studies^42–45^ conducted between 1999 and 2015 on surgical NEC in extremely preterm infants with a birthweight of less than 1000 g, overall mortality rates ranged from 38 to 59%. A recent international systematic review and meta-analysis^18^ on contemporary NEC outcomes found that the overall mortality rate in surgical NEC ranged from 35% (regardless of birthweight) to 51% (in infants with a birthweight < 1000 g). The findings of the present study in a Dutch nationwide population are in agreement with these figures, with a 30-day mortality of 47.2% in this specific, most vulnerable group of extremely preterm infants born at < 26 wGA.

When infants diverted to comfort care before surgery were included in the present analysis, the mortality rate among infants with severe NEC born at < 26 wGA was 63.9%, compared with 50% in the < 26 wGA subgroup of a previous nationwide study from the UK^20^. In that study^20^, the mortality rate was lower (21%) before laparotomy (comfort care) for severe NEC in infants born at < 26 wGA compared with the rate in the present study (31.9%). The UK study^20^ did not specify the number of infants who continued active treatment *versus* palliative care among those who did undergo laparotomy, where NEC totalis may be encountered frequently^26^. Nevertheless, differences in attitudes towards palliation and NEC totalis between countries and cultures may explain the differences in mortality rates^15,46^. In the present study, a relatively high percentage of infants had perforated NEC (56.3%) and pneumatosis intestinalis (65.5%) compared with other extremely preterm cohorts (35–54 and 29%, respectively)^47,48^, which may be indicative of more severe disease with an unfavourable prognosis in the present study population^49^. The overall high mortality risk highlights the relevance of the secondary aims of this study, namely understanding the underlying reasons for death, the impact of NEC totalis, and factors predictive of early postoperative mortality.

In the present study, 37 patients (26.1%) underwent open–close procedures, which entailed no further surgical treatment owing to NEC totalis. The definition of NEC totalis varies in the international literature, but is often not specifically defined^50^. Therefore, in the present retrospective study, the definition of NEC totalis was based on practical considerations: an open–close procedure in the case of massive bowel necrosis^26^. The number of open–close procedures provides a relevant overview of the proportion of infants born at < 26 wGA in whom Dutch surgeons deemed the NEC too extensive for a reasonable prognosis regarding quality of life. Although NEC totalis is not always reported in international surgical NEC cohorts, some other studies^1,7,26^ have reported rates comparable to those found in the present study, ranging from 14 to 27%.

It should be noted that decisions regarding diversion to palliative care (open–close procedure) in NEC totalis are based on considerations by the medical team and take into account parental preferences and ethical considerations, which may be culturally and regionally bound^15,46^. Yet, it may be tentatively concluded that the risk of NEC totalis in this select cohort of infants born at < 26 wGA is not much worse than that of older NEC populations. Although this is encouraging for this critical population, NEC totalis remains an undesirable outcome in any infant. Identifying those most at risk of NEC totalis continues to be challenging^26^. The future development of validated prediction scores and biomarkers is paramount to preventing unfortunate open–close laparotomies in NEC totalis.

In the present study, the 30-day non-lethal major complication rate was 21.9% in infants overall and 21% for those who survived to 30 days, consisting mostly of multiorgan failure or gastrointestinal sequelae, such as bowel perforations, anastomotic leaks, and anastomotic strictures that may lead to unplanned reoperation. The incidence of complications is similar to the reported rates of gastrointestinal sequelae in previous international NEC studies that also included infants born at a higher gestational age^12^. Interestingly, two Dutch cohorts^1,7^ in infants with surgical NEC born at < 32 wGA reported rates of surgical complications (30%) and unplanned relaparotomies (12%) that were comparable to the rates for the cohort born at < 26 wGA in the present study. With regard to intestinal failure, which is a relevant complication occurring beyond 30 days after surgery, the rate in the present study (27%) was higher than that reported by an international meta-analysis (13%)^12^, but slightly lower than that reported for a recent Dutch cohort (33%) of less preterm infants^7^. In summary, extremely preterm infants born at < 26 wGA treated surgically for NEC do not seem to be at significantly higher risk of developing non-lethal major complications than less preterm infants, supporting a relatively favourable postoperative course in these vulnerable infants—provided they survive the surgery. However, it should be kept in mind that the multifactorial causes of death (CM V) that occur in this critically ill population were not included in the major complication rate in the present study.

The significant 30-day postoperative mortality burden in the population of infants born at < 26 wGA population highlights the need for improvements in prediction, prevention, and outcome. Other studies on infants with surgical NEC of any gestational age have reported peritoneal drainage, cardiovascular support (during NEC or at the time of surgery), sepsis and blood transfusion within 48 h of surgery, lower gestational age, pneumoperitoneum, and panintestinal involvement as independent risk factors for 30-day mortality^1,37,38^. Despite careful selection of variables based on the available literature and clinical relevance, logistic regression analysis in the present study failed to identify any statistically significant preoperative risk factors for 30-day mortality. In addition, there was an absence of significant improvement in surgical outcomes over the 15-year study period. This highlights the well known and persistent difficulty of choosing between surgery and comfort care, with little predictive support in this critical population. In the future, accurate and group-specific outcome data, both short term and long term, will be all the more relevant for preoperative counselling and decision-making regarding these infants.

In the present study, the reported rate of diversion to comfort care (22.9%) in the full NEC stage ≥ II population provides a unique opportunity to better compare the mortality rate with that in international populations. A study from the UK^45^ reported that 4 infants (5%), from a population of 86 infants born at < 26 wGA with any emergency laparotomy indication, were diverted to comfort care before surgery. In another Dutch study^7^ of surgical NEC in infants with a birthweight < 1500 g, 28 of 135 infants (21%) were diverted to comfort care before surgery. Compared with these figures, the number of infants receiving comfort care in the present study seems to be on the higher side, although this does not seem to be specifically related to the prematurity of the population but rather country/culture-specific factors. This may result in country/culture-dependent selection bias towards fitter infants undergoing surgery. Therefore, future NEC studies should include these numbers for context and international comparison. Moreover, comfort care numbers are important for preoperative counselling and shared decision-making when NEC surgery is being considered for these infants. Recent developments in artificial intelligence in paediatric surgery focus not only on predicting NEC surgery but also on decision support regarding the choice of NEC surgery *versus* comfort care^15,51^, underscoring the importance of these clinical data.

The present study analysed a relatively large sample of a rare study population: infants born at < 26 wGA who underwent surgical treatment for NEC. The comprehensive assessment of the short-term postoperative course and surgical outcomes over time can provide relevant new data for counselling and the development of guidelines regarding NEC surgery in extremely preterm infants. The added comfort care context allows for future extrapolation to other study populations and a fair representation of the selection bias in this surgical population. This may prompt relevant studies on the origins of selection bias in surgical NEC for extremely preterm infants.

The results of the present study are limited by its retrospective design, leading to the historical loss of some data, as reflected in the incomplete comfort care numbers. Radiological images were not reassessed retrospectively, limiting the reliability of reported radiological findings. It should also be noted that typically there is confounding by indication, wherein the relatively most fit infants with NEC undergo surgery as opposed to diversion to comfort care. This may influence the generalizability of the results reported herein. There is no international consensus regarding the definition of NEC totalis and when to operate on these infants, making it challenging to accurately assess how many infants with massive bowel necrosis were diverted to comfort care, before or during surgery, compared with other cohorts. Finally, long-term outcomes were beyond the scope of the present study but are equally important in medical decision-making in NEC surgery.

In conclusion, infants born at < 26 wGA treated surgically for severe NEC were found to have a 30-day mortality rate of 47.2%. In more than half these infants, surgical treatment was limited to an open–close procedure because of the presence of massive bowel necrosis. In infants who did not undergo an open–close procedure, 21.9% experienced non-lethal major complications within 30 days, a rate comparable to that in the less preterm infants. Overall, the short-term outcome of NEC surgery in these extremely preterm infants—provided they survived the surgery—is not necessarily worse than that in more mature infants. This supports the rationale for undertaking surgical intervention as opposed to comfort care in selected patients when the clinical stability of the infant permits intervention. Regardless of the limits imposed by the clinical situation, the results of the present study can assist with future counselling, guideline development, and (shared) decision-making for infants born at < 26 wGA with an indication for surgery in the active phase of NEC. Future studies should focus on long-term outcomes in this vulnerable population.

## Data Availability

Anonymous data that support the findings in this study will be made available upon request to researchers who provide a methodologically sound proposal for use in achieving the goals of the approved proposal.
